# The Royal Infirmary, Preston

**Published:** 1914-10-03

**Authors:** Henry Burdett


### REPORTS ON

### Hospitals of the United Kingdom.

By SIR HENRY BURDETT, K.C.B., K.C.V.O.

**SERIES III.**

### THE ROYAL INFIRMARY, PRESTON.

This is an important, and in many ways one of the most interesting, county hospitals in Great Britain. It is situated rather outside the ordinary reach of those interested in hospitals, and has so failed, in our judgment, to receive that attention which its efficiency entitles it to receive at the hands of all interested in our voluntary hospitals. It was founded so recently as 1869, and it has had additions made to it on five subsequent occasions, commencing in 1876 and ending in 1910. It contains 158 beds, the average number occupied being about 120. The in-patients in 1913 numbered 1,770; 1,120 operations were performed, and general anæsthetics were administered 1,190 times. The death-rate was only 4.9 per cent. in 1913, it having been 7.1 in 1911, and 5.4 in 1912. In striking contrast to these results we may mention that the death-rate among the home patients in 1911 was 3.9; in 1912, 4.8; and in 1913, 13.6 per cent. The casualties amounted to 1,820, the general out-patients to 802, and home patients to 316; 766 operations were performed upon out-patients.

### Equipment and Bedding.

Preston has attained an extraordinarily high standard of efficiency. So well is it kept and maintained that it is literally true to say that there is not a square inch of any portion of the hospital that we could discover which is not attractive and well-kept enough to induce anyone to take their meals from it without any hesitation or doubt. The managers have deliberately laid down linoleum throughout the passages and many parts of the institution. This linoleum has stood twelve years' wear and tear, and it looked at the time of our visit as if it had only recently been laid. We have always felt ourselves that linoleum has disadvantages and possibly dangers, though the latter have not yet been demonstrably proved. Still, it presents considerable advantages, and if anyone has any doubt on that subject, they had better go to the Park Hotel at Preston, stay the night, and inspect with care the Preston and County of Lancaster Queen Victoria Royal Infirmary.

It must be borne in mind that the original hospital was built in 1869, and although considerable additions have been made practically during every decade since, the results as a whole are remarkably good. We did not see anything in the building to complain of; there was perfect order and cleanliness everywhere, and the equipment was good, with the exception of some of the bedding. It is astonishing that a hospital such as we have here described should still have flock, either for beds, pillows, or bolsters. The Report of the Inspectors of the Local Government Board on Flock, a copy of which we shall be glad to send to the authorities if it is not already in their possession, would convince the Committee, we are certain, that not a moment should be lost in substituting everywhere horsehair for flock throughout this hospital. The ward tables were specially good, and the efficiency of the domestic management, and the zeal and care of the matrons and sisters, were made strikingly apparent by the excellent cupboard provision, the most efficient ice-chests, and the general arrangements, which exhibited great method and thoroughness in all departments. So much was this the case that we took some trouble to satisfy ourselves that it was not veneer, but something much deeper and better. And there is no system so convincing as a process of questions and answers. These are the test of knowledge, and in this way a trained inspector can demonstrate the thoroughness or otherwise of the administration. Anything more striking or remarkable than the contrast in atmosphere, in the whole attitude and method of proceeding with their work, which characterised the staff of this hospital at Preston and that of a hospital not sixty miles away which we have recently reported on, it would be impossible to discover. At Preston Sister Gibson has been in charge of the same wards for twenty years. She is an admirable type of the high standard of the nurses who were trained before the present elaborate system, now so popular, had been widely introduced. She has her heart and soul in her work; she is happy as a queen, she has all the people under her happy too, and we are bound to believe that it is a privilege, if it could not in some sense even be called a joy, for a sick person to find himself, when ill in bed, under this sister's care. There are throughout our voluntary hospitals many such devoted women, and whenever we have the privilege of meeting with them we feel lifted up, and our hearts are stirred with gratitude that the sick throughout these islands are blessed by having such attendance in the hour of their special need and suffering.

### The Operation Theatre.

The operation theatre at Preston has its own special and peculiar features. The roof is semicircular, there is ample air and space, the ventilation is the best of any theatre we have seen in this country or abroad, and it seemed to us (and the surgeons confirmed our view) that from their point of view it is well nigh ideal. This theatre is rather larger than is now usual, but no one can doubt its excellence when they test it by the work done, the results, and the testimony of the surgeons who habitually use it.

The hospital contains an extraordinarily well-equipped and ample light and electrical department. There are few hospitals which possess anything like as good an equipment as that at Preston. It was a pleasure to inspect it, for it had "efficiency" written all over it. We hope, and we are assured, that its advantages are widely known in the district, and that the staff is so constituted as to enable the greatest amount of really good work to be done which undoubtedly could be done, and is being done, here, judging from the figures contained on page 7 of the Report of the Board of Management for 1913. At the time the original part of the hospital was erected it was customary to put the fever hospital on a contiguous site. Nowadays hygiene has a better chance of teaching the lessons the enforcement of which tends to save the lives and to improve the health of the citizens. Now, the old fever hospital has been converted for the reception of patients in connection with certain special departments where the work is proceeding with efficiency.

There is a mortuary which should be modernised by the addition of a view-room or chapel. The pathological department is good, and constitutes another proof of the thorough way in which the medical and surgical work is carried on at this institution. There is a nurses' home which might be improved by the addition of a nurses' library of good books, and we thought the recreation sitting-rooms for the nurses could be made infinitely more comfortable by the addition of some chairs, the chief feature of which is the comfort they give to the sitters who occupy them. This is a point which seems to us to be continually overlooked by those who are responsible for the furniture of nurses' homes. The latest and most remarkable example of that is the Children's Hospital at Glasgow, which was recently opened by their Majesties the King and Queen.

### Some Fundamental and Business Matters.

The authorities of the hospital have not been well treated by the Corporation of Preston, who have withheld their subscription of 50 guineas because the treatment of the school children at Preston has not been specially provided for at the infirmary. We are of opinion that the greater voluntary hospitals ought not to have cast upon them the cost and heavy work entailed by the treatment of the school children of a large city. Every municipality ought to have its own school clinic or clinics, and to provide the cost of this treatment by an arrangement with the medical profession. We would, however, suggest, in view of the probable modifications which will have to be made in the present provision for and rules of admission of in-patients to voluntary hospitals, that Preston might usefully follow the example of the authorities of the Leicester Infirmary, where the municipal, county, and health authorities have by arrangement agreed to appoint representatives to serve on the County Infirmary Board. In striking contrast to the Corporation of Preston, we note with pleasure that the Board of Guardians there have increased their annual subscription from £25 to £50 a year.

As testifying to the careful management, we observe that the ordinary expenditure of 1913 shows a decrease of £246, there being in the same year an increase in the annual income. Still, the Preston Royal Infirmary needs an additional income of £2,500 a year, and, in view of what we found during our visit, and have here had the pleasure to describe, we should hope that the whole of this revenue will be provided by the citizens of Preston before the end of 1914.

### Finance.

It may be interesting to record how the money for some of the extensions of the Royal Infirmary, Preston, was raised. Upwards of £42,000 was raised, as to £7,800 as a Diamond Jubilee Fund, and the balance as the Preston Memorial of Queen Victoria's reign. As the result of an issue of 12,000 appeals, without any personal canvass of any kind, the money actually received exceeded the total expenditure by £2,539. This is undoubtedly a record, and we congratulate the board of management and their Secretary, Mr. W. Davies, and also the medical and resident staff of the Royal Infirmary, Preston, one and all, for they all have contributed to the successful accomplishment of this remarkable result. First, there was the undoubted efficiency throughout the administration and management of this hospital; then there was the interest taken by the business men of Preston and the pride they deservedly take in their hospital; then there was the interest, care, and continuous control exercised by Mr. Dixon, the architect. All must have put their heart into the good work, and all deserve their meed of congratulation and thanks.

The works to which we have just referred were completed in 1904. Since then the *x*-ray department, a sterilising room, and observation wards have been added, and in addition the kitchen has been thoroughly overhauled, reconstructed, and equipped. The extra cost of these works has been raised by private subscription, without the issue of any general appeal. Finally, we wish to express our acknowledgments to the members of the honorary staff, especially to Dr. Collinson, together with the Matron, Miss M. A. Marks, and the Assistant Matron, Miss A. Charlesworth, whom we had the pleasure of meeting during our inspection.

